# *Mycobacterium avium* subsp. *hominissuis* Infection in a Domestic Rabbit, Germany

**DOI:** 10.3201/eid2403.171692

**Published:** 2018-03

**Authors:** Daniela Klotz, Stefanie A. Barth, Wolfgang Baumgärtner, Marion Hewicker-Trautwein

**Affiliations:** Author affiliations: niversity of Veterinary Medicine, Hannover, Germany (D. Klotz, W. Baumgärtner, M. Hewicker-Trautwein);; Friedrich-Loeffler-Institut/Federal Research Institute for Animal Health, Institute of Molecular Pathogenesis, Jena, Germany (S.A. Barth)

**Keywords:** *Mycobacterium avium* subsp. hominissuis, enteritis, rabbit, bacteria, zoonoses, Germany

## Abstract

*Mycobacterium avium* subsp. *hominissuis* is an opportunistic pathogen present in soil and dust. We report *M. avium* subsp. *hominissuis* infection found in a domestic rabbit in Hannover, Germany, in May 2017.

*Mycobacterium avium* subsp. *hominissuis* is an opportunistic pathogen with zoonotic potential (*1*,*2*) that is present in soil and dust. Animals are seen as a reservoir and potential threat for human infection, but the route and source of human infection remains unknown in most cases (*3**–**6*). We report on a 4-year-old intact male rabbit from a private breeder in Germany that died suddenly in May 2017. Several months before death, the rabbit showed intermittent diarrhea, and the veterinarian suspected coccidiosis. Necropsy findings included cachexia with small amounts of a clear fluid in body cavities due to hypoproteinemia; dehydration; multifocal intramural nodules <4 mm in diameter in the jejunum and ileum; highly liquefied intestinal contents without molding; and enlarged mesenteric lymph nodes. We detected single coccidia parasites in a native intestinal smear. Histologically, the nodular lesions in the ileum showed severe granulomatous enteritis with large areas of necrosis and numerous multinucleated giant cells (Figure, panel A). Ziehl-Neelsen stain demonstrated large numbers of acid-fast bacilli in macrophages and multinucleated giant cells in the intestine (Figure, panel B). The mesenteric lymph node also exhibited a granulomatous inflammation with multinucleated giant cells. Additionally, the rabbit had mild suppurative splenitis, mild lymphohistiocytic to granulomatous hepatitis, mild focal lymphocytic interstitial orchitis, and a hyperplasia of the myeloic cell line in the femoral and sternal bone marrow. We detected no acid-fast bacilli in the mesenteric lymph nodes, the spleen, or the liver.

**Figure:**
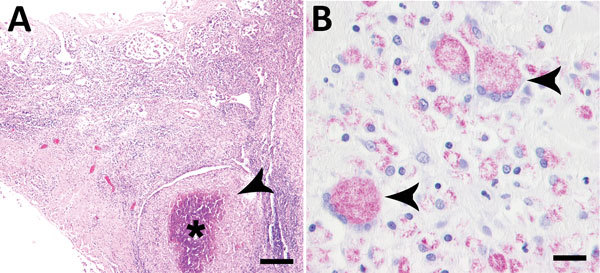
Results of histologic testing of samples from a domestic rabbit with *Mycobacterium avium* subsp. *hominissuis* infection, Germany. A) Hematoxylin and eosin stain reveals multifocal severe granulomatous enteritis in the ileum with focally extensive necrosis (asterisk) and numerous surrounding macrophages (arrowhead). Scale bar indicates 300 µm. B) Ziehl-Neelsen stain shows numerous acid-fast bacilli in the cytoplasm of macrophages and multinucleated giant cells (arrowheads). Scale bar indicates 20 µm.

We decontaminated sections of the small intestine using NALC-NaOH and cultivated on Löwenstein-Jensen (Artelt-Enclit GmbH, Germany), Stonebrink (Artelt-Enclit), and Herrold’s Egg Yolk (Becton Dickinson GmbH, Germany) agar slants (the last supplemented with Mycobactin J), as well as in Kirchner medium (Artelt-Enclit). We extracted DNA from grown colonies after heat inactivation by ultrasonic cell lysis and analyzed the DNA by PCRs targeting insertion sequence (IS) *1245*, IS*900*, and IS*901*. The presence of IS*1245*-specific and absence of IS*900*- and IS*901*-specific PCR products identified the bacilli as *Mycobacterium avium* subsp. *hominissuis*. Additionally, DNA sequencing of a *rpoB* gene PCR fragment yielded 100% sequence identity to *rpoB* from *M. avium* ssp. *hominissuis* strain IWGMT49 (GenBank accession no. EF521911) (*7*).

*M. avium* subsp. *hominissuis* is not currently a reported pathogen for rabbits. It has been reported only once in a slaughtered rabbit, but that animal showed no clinical or pathological abnormalities (*8*). In our investigation, as in reports in other host species, the source and route of infection was unclear. The presence of enteric inflammatory lesions with presence of acid-fast bacilli, however, suggests an oral route of infection.

It has been reported that a mycobacterial infection is dependent on the immunity and nutritional status of the host (*2*,*9*). In this case, the infestation with coccidia, common intestinal parasites in rabbits that can cause emaciation, may have contributed to the massive mycobacterial infection. Nevertheless, clinically manifest mycobacterial infection is a rare finding in domestic rabbits. We encourage awareness of a potential zoonosis, such as infection with *M. avium* subsp. *hominissuis*, in rabbits with intermittent diarrhea and chronic weight loss.
